# Main Techniques to Reduce Concentrate and Achieve Salt–Organic Separation During Landfill Leachate Treatment Using Low-Rejection Nanofiltration Membranes

**DOI:** 10.3390/membranes15100308

**Published:** 2025-10-10

**Authors:** Alexei Pervov, Dmitry Spitsov, Tatiana Shirkova

**Affiliations:** Department of Water Supply, Moscow State University of Civil Engineering, 26, Yaroslaskoye Highway, 129337 Moscow, Russia; spitsovdv@mgsu.ru (D.S.); mabell@bk.ru (T.S.)

**Keywords:** reverse osmosis, nanofiltration, membrane rejection, low-rejection membranes, landfill leachate, concentrate utilization

## Abstract

Landfill is a source of environmental concern as it may contaminate surface and groundwater, which could be a major source of potable water supply. Reverse osmosis (RO) membrane treatment is a well-known technique for treating leachate, but it requires high pressures of 80 bars or more to function. In addition, pretreatment, scaling, biofouling and concentrate disposal bring additional challenges to RO operation. The use of nanofiltration (NF) membranes with low rejection ensures the concentrate is separated into organic and salt solutions at a low pressure of 16–18 bars and ensures the concentrate volume is reduced to less than 3% of its initial value. This results in a reduction in energy consumption by a factor of least three compared to using conventional high-pressure RO, which reduces the initial leachate amount to 9–10%, and evaporation results in a subsequent reduction in concentrate volume to 3–4 per cent of the initial leachate volume. Due to the low osmotic pressure, the volume of an organic solution after separation can be reduced by three to four times compared to a saline solution of the same concentration.

## 1. Introduction

Landfill leachate is a highly complex solution that causes concern among researchers. The chemical composition of landfill leachate is usually distinguished by extremely high COD (chemical oxygen demand) and TDS (total dissolved salts) values [1,2,3,4,5]. Current technologies to treat, purify, and reduce the volume of leachate include the following:

- Physical and chemical methods [3,4] based on adsorption, aeration, floatation, coagulation, flocculation [4,5,6,7,8,9,10,11], chemical deposition, and membrane separation methods (ultrafiltration, reverse osmosis, nanofiltration [6,9,10,11]), as well as chemical oxidation methods [4];
- Biological methods of wastewater treatment that include aerobic and anaerobic treatment and “annamox” methods [8];
- Evaporation methods that include heating techniques as well as steam compression techniques [12,13,14].

The described approach to treat leachate appears to be very complicated as it contains many procedures to solve different tasks:

- Pretreatment: Removal of suspended matter and organics that form COD and BOD [11,15,16]. The use of flocculation chemicals [14], sedimentation tanks, and flotation tanks, as well as bioreactors [15,16], seems “excessive” but is dictated by the concern of membrane fouling [17].
- The chemical step—removal of calcium—is considered necessary, as membrane modules suffer scaling problems, especially when recovery is increased [5,11].
- The high-pressure step also provides limited recovery as the concentrate is reduced to 10 percent of its initial value [13,18,19,20,21,22,23].

Reverse osmosis (RO) has some disadvantages connected with membrane fouling and high operational costs when handling and utilizing RO concentrate streams [6,7,9,10,11].

The concentrate is utilized during its evaporation, incineration, and oxidation, but these methods require high investment. One way to reduce concentrate volume is to use ultrahigh-pressure RO membranes [13].

To further reduce concentrate flow, an additional reverse osmosis stage is used that is operated under a pressure of 140 bars [13]. This stage uses specially designed spiral wound membrane elements that can withstand high pressures. The use of an additional RO high-pressure stage, reduces the concentrate flow to 10 percent of the initial leachate flow. This high-pressure membrane stage is three times more energy-efficient than evaporation.

Evaporation as a method of utilizing reverse osmosis concentrate cannot be considered effective [13,14]. Landfill leachate cannot be completely evaporated to the state of “dry salts”, mainly because of the high content of organics that act as an inhibitor of crystallization, the complexity of the evaporation system design, and high capital and operational costs (as well as high operational costs that require 23 kWh to evaporate one cubic meter of concentrate). The minimum volume achieved during a study of evaporation experiments was no less than 4 percent of the initial volume of leachate [12,13].

The authors of [13] provide data for an economic comparison of membranes and evaporation used for reducing RO concentrate.

To reduce and utilize this concentrate after seawater desalination, membrane-based technology would need less than half of the energy consumed by conventional thermal evaporation technology. To increase brine TDS from 70,000 to 250,000 mg dm^−3^ using evaporation, the estimated specific energy consumption would be 24 kWh m^−3^. Such a concentration could be achieved by using the two-stage high-pressure RO at a lower power consumption of 7.3 kWh m^−3^. The maximum operational pressure for typical seawater RO is 80 bars and, to achieve a concentration of 250,000 mg dm^−3^, a pressure of 300 bars is required (the osmotic pressure of the solution with a salt content of 250,000 mg dm^−3^ is around 290 bars). Treatment of landfill leachate with 15,000 ppm of TDS and 20,000 ppm of COD with high-pressure RO membranes also requires a pressure of 70–80 bars and can only provide a three-fold reduction in leachate volume. To further reduce this volume by two or three times, special membranes operating at a pressure of 120–140 bars are used. The concentrate volume is further reduced to a value not exceeding 2.5–3% of the initial volume of leachate using evaporation.

Many years of research have prompted us to propose more simplified and less expensive solutions to achieve the main goals of leachate treatment: to reduce the concentrate flow and find ways of utilizing it completely; to reduce the cost of power needed to treat and concentrate leachate; and to solve the problems of pretreatment and fouling control. These solutions were developed over 30 years in a number of publications presented at a number of EDS and IDRA conferences since 1991 [18,19,20,24,25].

**Figure 1:**
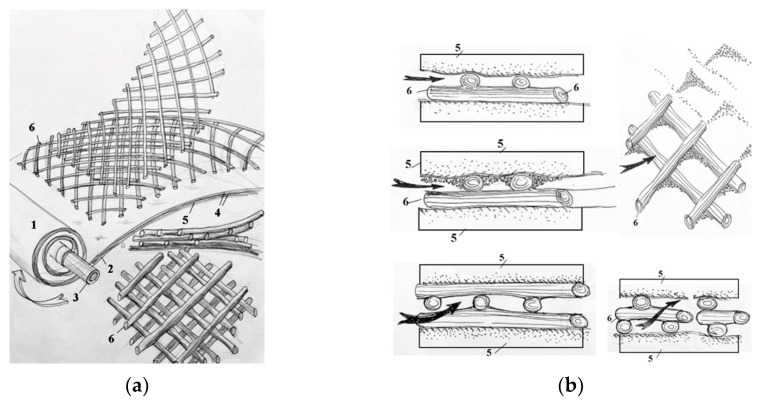
Components of “open channel” formation during the manufacture of the spiral wound element: (**a**)—formation of the “open channel”; (**b**)—formation of the living cross-sectional area and its performance during colloidal fouling: 1—membrane envelope consisting of two membrane leaves glued together; 2—outer edge of the glue; 3—tube for permeate collection; 4—membrane; 5—permeate carrier tricot between membrane leaves; 6—spacer mesh.

The use of membrane elements with an “open channel” on the first membrane stage [24,25] simplifies pretreatment and allows colloidal and organic foulants to remain during this stage, as they can be easily removed by applying hydraulic flushings due to the high living cross-sectional area of the flow in membrane channels and, consequently, less hydraulic resistance to the flow even after fouling. Components of “open channel” formation during spiral wound membrane production are presented in Figure 1a,b.

**Figure 2:**
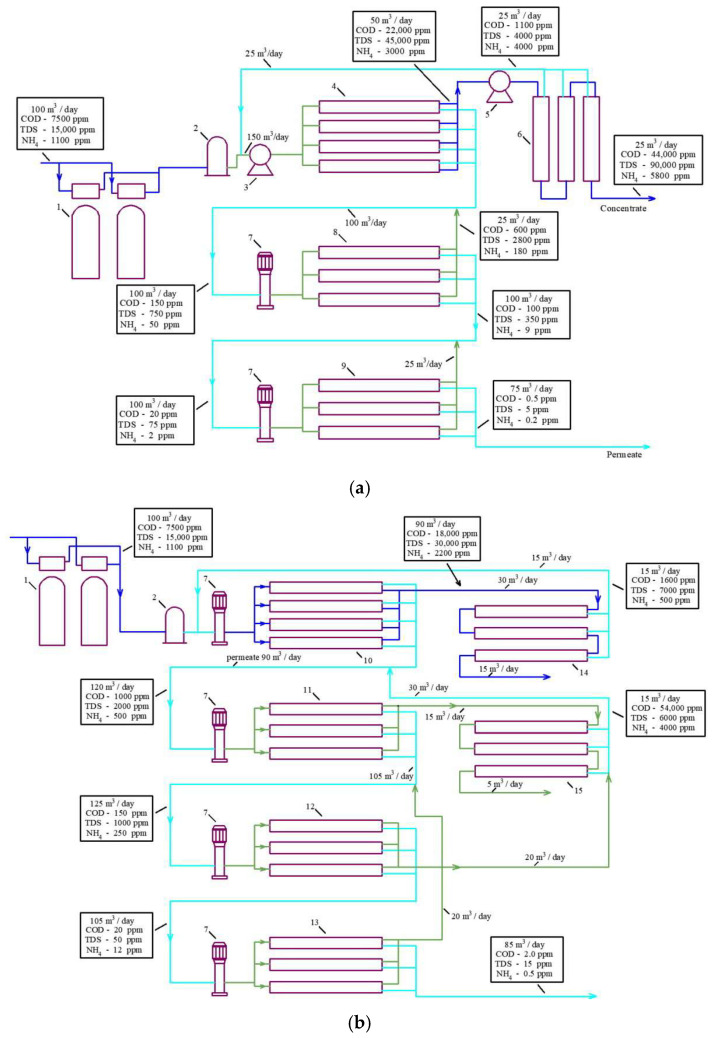
Flow diagrams of reverse osmosis membrane facilities used to purify landfill leachate: (**a**) technology that uses high-pressure “seawater” reverse osmosis membranes with an additional “super-high-pressure” membrane stage; (**b**) a new approach to reducing concentrate: the use of low-pressure low-rejection nanofiltration membranes to increase recovery. 1—pretreatment sand filters; 2—pretreatment disk microfilters; 3—high-pressure piston working pump; 4—first stage of high-pressure RO membranes (“seawater” membranes); 5—high-pressure pump; 6—extreme-high-pressure membranes (spiral wound modules or plate-and-frame modules); 7—second and third stages of working centrifugal pump; 8—second stage of low-pressure RO modules; 9—third stage of RO membrane modules; 10—first stage nanofiltration membranes; 11—first stage low pressure RO membranes; 12—second stage RO low pressure membranes; 13—third stage RO low pressure membranes; 14—second stage of low rejection nanofiltration membranes to increase recovery of the first nanofiltration stage; 15—second stage of low rejection nanofiltration membranes to increase recovery of the first RO stage.

Figure 2a describes a conventional approach to treating landfill leachate using high-pressure and high-rejection reverse osmosis membranes usually applied in seawater desalination projects. The use of “seawater” high-pressure membranes provides high-quality water but fails to obtain a concentrate flow rate less than 30% of the initial leachate fed into the reverse osmosis facility [9,10]. Additional super-high pressure membrane modules are used to reduce the concentrate flow rate to 10 per cent of the initial feed water flow rate.

Figure 2b presents a scheme for facilitating landfill leachate treatment with nanofiltration membranes [18,19], which allows a high concentration ratio of the initial leachate to be achieved by applying a “cascade” of nanofiltration membrane modules [13,18,21,22,26].

The principle of “osmotically assisted RO” is to reduce the osmotic pressure gradient across the membrane by allowing brine of a certain salinity to flow on the conventional permeate side of the membrane [13]. The feed-to-reject side of the membrane is pressurized while the permeate side has a much lower pressure, and if the pressure difference between these two sides is higher than the osmotic pressure difference, water molecules will pass through the semi-permeable membrane. In other words, to pass the permeate through the membrane, we need to generate a pressure higher than the difference between the pressure in the concentrate and permeate. This principle is the basis of new technologies used to concentrate seawater [2] and used in our research to reduce the concentrate formed after landfill leachate treatment. The flow diagrams presented in Figure 2 demonstrate the TDS, COD, and ammonium concentration in concentrates and permeates in all leachate treatment stages.

To achieve an efficient reduction in ammonium and volatile organics, at least two RO membrane stages are used. Figure 2a,b demonstrate additional membrane stages that use low-pressure RO membranes with a rejection of 96–99 to remove ammonium and volatile organics. Ammonium concentrations in leachate can usually reach 1000–2000 ppm, so we need at least three RO stages to reduce the concentration of ammonium by 5000–10,000 times to reach regulation values of 0.2–0.5 ppm for ammonium ion discharge in natural sources. Each RO stage reduces ammonium by 30–50 times depending on pressure and recovery. The first stage (seawater high-pressure membranes) reduces the ammonium ion concentration by 50 times. In the second stage, the concentration of ammonium is reduced by a factor of 50 × 30, or 1500. The third stage provides an additional reduction by 30 times; thus, the overall decrease in ammonium concentration can be 50 × 30 × 30, that is, 45,000 times. The flow diagrams shown in Figure 2 and take into account the decrease in ammonium concentration in each stage.

The main disadvantage of existing technologies appears to be the use of high-pressure membranes developed for seawater desalination. The application of low-rejection nanofiltration membranes [27,28] and the “staged” technique of their use in leachate treatment [18] enables us to escape scaling [24], to reduce concentrate, and to increase concentrate TDS up to 150,000 ppm and higher using “the osmotically assisted RO” [13] at pressures no higher than 16–18 bars [18,19].

The use of nanofiltration membranes in many cases excludes the formation of scale in spiral wound modules [24]. Modification of the spiral wound membrane channel and the use of the “open channel” provides reliable operation to reduce hydraulic resistance, as well as simplifies pretreatment [12,25,29,30,31].

The presence of organics creates additional osmotic pressure that complicates leachate treatment and concentrate flow reduction. The separation of organics and salts and the separate treatment and concentration of these concentrate flows provide an efficient reduction in total concentrate volume without additional power consumption. The selection of membrane characteristics (difference between rejection of monovalent ions and organics) is very important for the efficient reduction in the total concentrate amount [25,26,32,33].

In this article, in addition to developing the concentrate reduction techniques, we aimed to demonstrate that colloidal and organic foulants as well as calcium carbonate scaling do not pose a serious threat to membranes and can be controlled by cleaning and flushing [24,25] instead of complicating the pretreatment scheme.

## 2. Materials and Methods

### 2.1. Research Methodology: Techniques to Further Reduce Concentrate Discharges by Separating Organics and Salts

The concentrate of reverse osmosis plants that treat landfill leachate contains dissolved organics that increase osmotic pressure and thus reduce permeability. Thus, the presence of organics in landfill leachate greatly influences operation costs [34,35,36]. Therefore, the separation of organics and salts enables us to substantially reduce the concentrate without additional power costs, as organic solutions can operate at an osmotic pressure 4–5 times lower than that of electrolyte solutions with the same concentration [25]. The techniques to reduce concentrate flow via its separation in organic and salt solutions are presented below.

Table 1 shows the chemical compositions of the landfill leachate solutions with different COD to TDS ratios used in the studies.

The “Aleksandrov” landfill leachate chemical composition is taken from Table 1 as an example, where the landfill contains 5000 ppm of COD and 4000 ppm of TDS. For salt solution with a TDS value of 4000 ppm without organics, we can achieve a salinity of 60,000 ppm in concentrate by applying high-pressure reverse osmosis membranes [13,32,33] and using a working pressure of 60 bars (Figure 2a). We can also apply a “cascade” of low-rejection and low-pressure nanofiltration membranes and reach a concentration of salts of 160–180 g per liter at a working pressure of 15–18 bars (Figure 2b). Thus, initial feed water salinity is increased by 15 and 40–45 times in the first and second cases, respectively (Table 2). Techniques to increase concentration using the reverse osmosis method are based on the assumption that the required pressure is evaluated not by the concentration of the separated solution but by the difference between osmotic pressure in the concentrate and permeate. The feed water solution (landfill leachate) with a salinity of 4000 ppm has an osmotic pressure of 3 bars, and the organic solution with a COD value of 5000 ppm has an osmotic pressure value of 0.85 bars. When the feed water with a salinity of 4000 ppm is concentrated 15 times, we reach 60,000 ppm in the concentrate, which corresponds to an osmotic pressure of 45 bars (Table 2). We apply a working pressure of 60 bars, reach an osmotic pressure of 45 bars, and provide a “driving force” (difference between the working pressure and osmotic pressure in concentrate) of 15 bars. For organics in the feed solution, after we increase the initial concentration by 15 times, we achieve an osmotic pressure of (3 + 0.85) × 15 = 57 bars that eliminates the “driving force” of the process. This explains why the presence of organics is an obstacle to reaching high recoveries.

The TDS of “Timokhovo” landfill leachate (Table 1) is 15,000 ppm, which allows the RO facility to reduce the concentrate to only 30 percent of the initial flow (Table 2). The presence of dissolved organics that provided a COD value of 20,000 increased the osmotic pressure by bars, which also substantially reduced the “driving force” and the final recovery value. This has prompted the “Timokhovo” landfill management to switch to the pressure scheme (Figure 2a).

In this article, a new technique is proposed that consists of separating the landfill leachate into two concentrated solutions, a salt solution and organic solution, which substantially reduces the concentrate discharge as the organic solution is better concentrated due to lower osmotic pressure. Table 3 shows the treatment of landfill leachate collected from “Voskresensk”. The COD value of leachate is 7500 ppm and its TDS value reaches up to 15,000.

**Figure 3:**
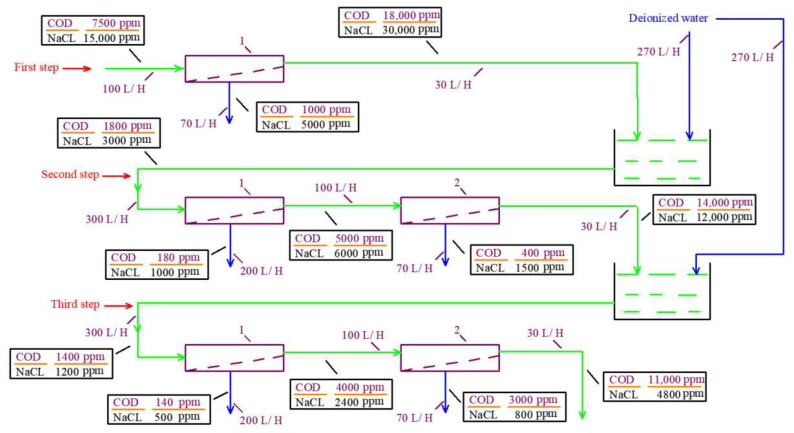
The flow balance diagram of the steps to separate the concentrate of the “Voskresensk” landfill leachate (Table 1) into organic and saline solutions and to increase the COD/TDS ratio. The TDS value is 4000 ppm and the COD value is 5000 ppm. First step: Concentration of the feed leachate; second step: dilution of the concentrate by 10 times with deionized water, with further concentration; third step: secondary dilution by 10 times with further concentration. 1—first stage low rejection nanofiltration membranes; 2—second stage nanofiltration membranes to reduce concentrate.

The chemical composition of “Voskresensk” landfill leachate is presented in Table 1. The COD value of leachate is 7500 ppm and its TDS value reaches up to 15,000 ppm. To separate organics from ions, low-rejection nanofiltration membranes are used with different rejection characteristics of organics (expressed by COD) and monovalent ions [29]. When the leachate passes through the nanofiltration membrane module, the ratio between the concentration of organics and salts (COD-to-TDS ratio) increases in the concentrate as the membrane rejects organics better than monovalent ions. In the permeate, the COD/TDS ratio is reduced after monovalent ions (chloride and sodium ions) penetrate better through the membrane. After we increase the COD/TDS ratio by 2–3 times during the first membrane pass (the first cycle), the concentrate can be diluted by deionized water to obtain the initial COD value in the mixture. Then, the second concentrating cycle can be applied and the COD/TDS ratio further increases due to the preferential passage of sodium and chloride ions through the membrane. The flow diagram of this process is presented in Figure 3. The third dilution and concentrating cycle provides a further increase in the COD/TDS ratio. Figure 3 shows the balance flow diagram of the three steps for increasing the COD-to-TDS ratio. The results of the osmotic pressure calculations in permeates and concentrates of membrane modules in each membrane stage are presented in Table 2. The calculated osmotic pressures of sodium chloride solution and organic solution are also presented in Table 3 for comparison as “solutions after separation”. Table 3 also presents the results of similar calculations performed for the “Timokhovo” and “Aleksandrov” landfills.

For the landfill “Voskresensk” (Table 1), the experimental test procedure and guidelines to modify the concentrate treatment technology were developed. Figure 3 shows the balance flow diagram of the three steps for increasing the COD/TDS ratio. The results of osmotic pressure calculations in concentrates and permeates in each membrane stage are presented in Table 3.

As calculating osmotic pressure is complex, we approximated it by making some assumptions. For seawater with a salinity of 30,000 ppm, the osmotic pressure is 25 bars. Assuming that the majority of ions are represented by sodium and chloride, we evaluated the osmotic pressure of sodium chloride solutions in proportion to the change in their TDS. The organic non-electrolyte solutions have an osmotic pressure that is on average one fourth of that of the NaCl solution.

**Figure 4:**
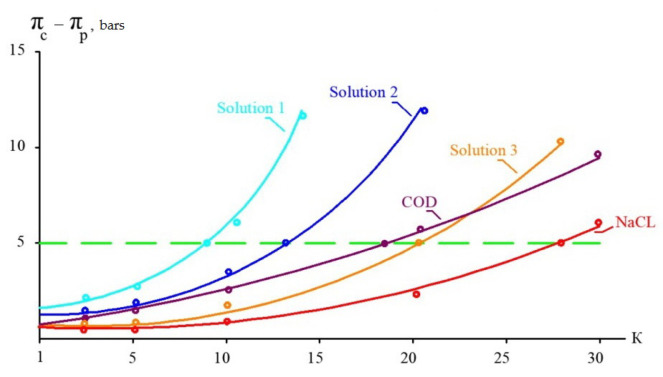
Difference between osmotic pressure in concentrate and permeate on K: solutions 1, 2, and 3 are shown in Table 3; model solutions of NaCl and COD after separation are shown in Table 3.

Table 3 presents the results of calculations based on the assumption that “70NE” membranes provide a 90 percent rejection of organics and 60–70 percent rejection of monovalent ions. Osmotic pressures of pure sodium chloride and organic solutions are also presented in Table 3 for comparison. Based on the results presented in this table, a graph to determine the maximum initial volume reduction coefficient K is presented in Figure 4. Figure 4 shows the dependence of the “driving force” (the difference between osmotic pressures in concentrate and permeate) on the K value, which are presented for the three steps of leachate treatment (Figure 3) and for pure solutions of sodium chloride and organics (Table 3). A horizontal line parallel to the abscissa axis shows the selected maximum difference in osmotic pressure that should not be exceeded to provide enough “driving force” for leachate purification using low-pressure, low-rejection nanofiltration membranes. The calculation results are shown in Figure 4 as graphs of the dependence of pressure, in bars, on the K value. The higher the difference between organics and monovalent ion rejection, the better the effect of separation. In our research, we compare the separation efficiencies of nanofiltration membranes of the “70NE” model (”Toray Industries, Inc.”, Tokyo, Japan) and a new membrane of the nanoNF model (JSC “Membranium-RM Nanotech”, Vladimir, Russia). The low rejection of NaCl by the “nanoNF” membranes and the high rejection of organics allowed us to achieve a substantial increase in COD-to-TDS by using only two cycles of concentration and dilution.

The goal of this study is to demonstrate a new technology to separate the concentrate into organic and salt solutions individually. The use of low-rejection nanofiltration membranes provides an efficient solution and the potential to achieve concentrations of TDS up to 70–100,000 ppm and COD up to 180,000 ppm. The efficiency of separation is dependent on the difference between the rejection of organics and monovalent ions. The greater the magnitude of this difference, the higher the separation effect and the higher the COD-to-TDS ratio achieved in the concentrate; this is shown below. The selection of the most effective membranes is the main result of our research. The article also describes another benefit of using modified “nanoNF” membrane modules with “open channels” that demonstrate small fouling propensities, simplifying pretreatment and reducing the concentrate. Low-rejection membranes make it possible to overcome the disadvantages of conventional technologies based on high-pressure membranes.

The main experimental goals of the experiment were as follows:

- Select membranes that are more suitable for solving the problems of handling and utilizing landfill leachate concentrate;
- Experimentally determine the dependence of the rejection of different substances and the product flow for different membranes;
- Separate organics and ions to produce concentrates with organics and with salts;
- Investigate the scaling process during landfill leachate treatment with RO and NF membranes;
- Investigate organic fouling;
- Evaluate the required membrane surface and the number of membrane elements in each stage;
- Develop the process of organics and salt separation for reducing concentrate flow.

### 2.2. Experimental Procedure: Materials and Equipment

**Figure 5:**
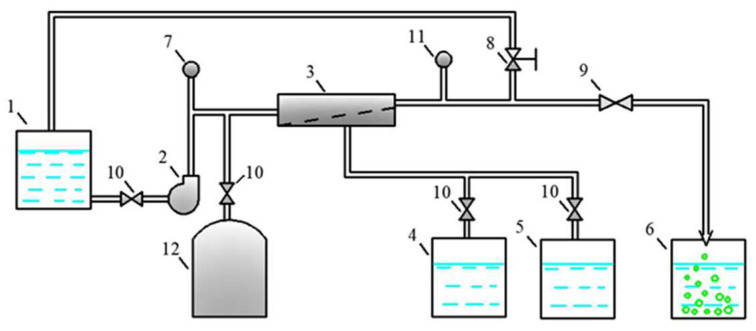
The flow diagram of the membrane test unit used in the experimental program: 1—feed water tank; 2—working pump; 3—membrane spiral wound module; 4, 5—permeate collection tanks to test permeate quality at different recoveries; 6—flush water collection tank; 7—pressure gauge at the outlet; 8—pressure regulation valve; 9, 10—valves; 11—pressure gauge at the inlet; 12—pressure tank to increase the transit flow.

Experiments to separate the leachate from the “Voskresensk” landfill (Moscow region) were conducted using the test membrane plant. The flow diagram of the test unit is shown in Figure 5. The leachate feed solution was placed in tank 1 and then directed using pump 2 to membrane module 3. The working pressure was 13-16 bars. Membrane modules of the 1812 standard (1.8 inches in diameter and 12 inches in length) tailored with nanofiltration membranes were used. “NanoNF” membranes were supplied by JSC “Membranium-RM Nanotech Company” (Vladimir, Russia) and “70NE” membranes were manufactured by ”Toray Industries, Inc.” (Tokyo, Japan). The modified experimental membrane element of the “nanoNF 1812-C” model demonstrated NaCl and MgSO_4_ solution rejections of 50% and 90%, respectively. The product flow was 17.8 L per hour, the test pressure was 8 bars, and the temperature was 25 degrees Celsius. The test solution with concentrations of NaCl and Na_2_SO_4_ of 500 ppm were used to test the membrane elements with “nanoNF” membranes. The membrane element of the “70 NE 1812” model demonstrated rejections of NaCl and MgSO_4_ solutions of 70% and 95%, respectively. The product flow was 14 L per hour. These elements were tested with 250 ppm solutions of NaCl and of Na_2_SO_4_. The test pressure of “70 NE” membrane elements was 4.1 bars and the temperature was 25 degrees (Celsius). Both elements were tested at 15 per cent recovery and had a membrane surface area of 0.5 square meter. The “NE 70 1812” element used a standard-size separator turbulization mesh with a thickness of 31 mils. The modified “nanoNF 1812” element contained an “open channel” with a spacer thickness of 47 mils.

Purified water (permeate) was collected in permeate tank 4, and the concentrate was returned to feed water tank 1.

Organics and monovalent ions were separated in three steps. In the first step, the leachate volume was reduced from 20 to 2 L (concentrated by 10 times).

Further, in the second step, the concentrate was diluted with deionized water produced by the treatment of Moscow tap water using a membrane spiral wound module (model “RE-1812 HR 75” manufactured by the company “Raifil”, Moscow, Russia). After the concentrate was diluted by 10 times, it was again placed in tank 1 and again concentrated by 10 times.

In the third stage, the obtained concentrate was again diluted by 10 times and further concentrated by 10 times. In each stage, the ratio of COD to total salt content (TDS) in the permeate and concentrate changed continuously due to the difference in membrane rejection for organic matter and salt ions. The permeate obtained after the third stage was a mixture of ammonium chloride and sodium chloride solutions with a salt content of about 2000 mg/L. Such a solution can be effectively concentrated using reverse osmosis and nanofiltration membranes up to a salt concentration of 150 g/L and higher [22,25].

Rotary-type pumps manufactured by “Fluid-o-Tech Asia (Shanghai) Co., Ltd.” (Shanghai, China) were used for working pressures of 12–16 bars.

The COD, alkalinity, hardness, and concentration of calcium and chloride ions were determined by volumetric (titrimetric) methods; the concentrations of ammonium ions and heavy metal ions were determined by photometric methods; the concentration of sulfate ions was determined by turbidimetric methods; and the specific conductivity was evaluated using conductometric methods.

## 3. Discussion of the Results

### 3.1. Experimental Dependencies

**Figure 6:**
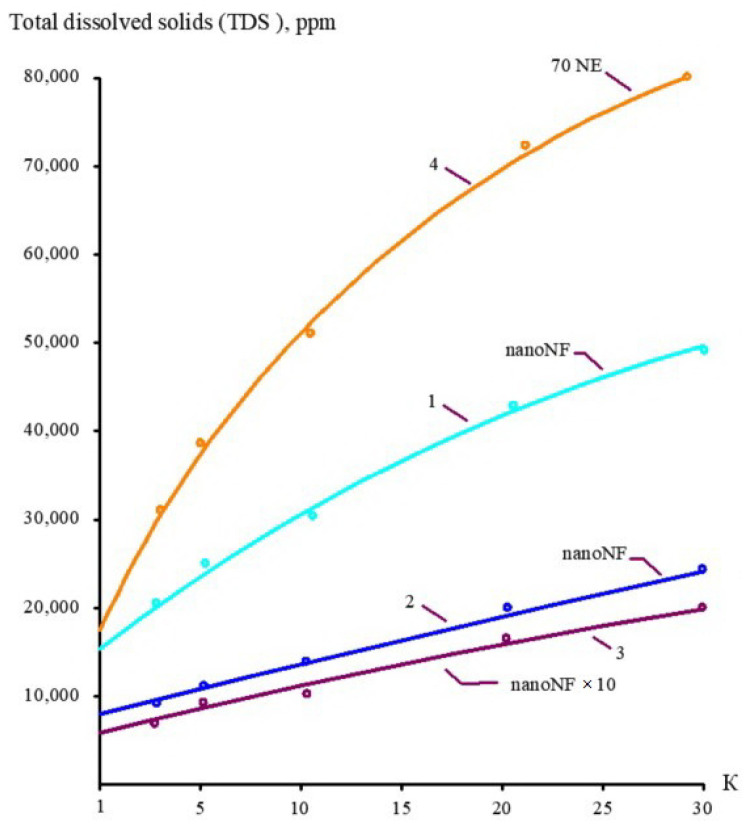
The dependencies of TDS in permeates and concentrates on K: 1—first stage of leachate concentration using “nanoNF” membranes; 2—first stage of permeate treatment using “nanoNF” membranes; 3—first stage of leachate concentration after dilution using “nanoNF” membranes; 4—first stage of leachate treatment using “70NE” membranes.

**Figure 7:**
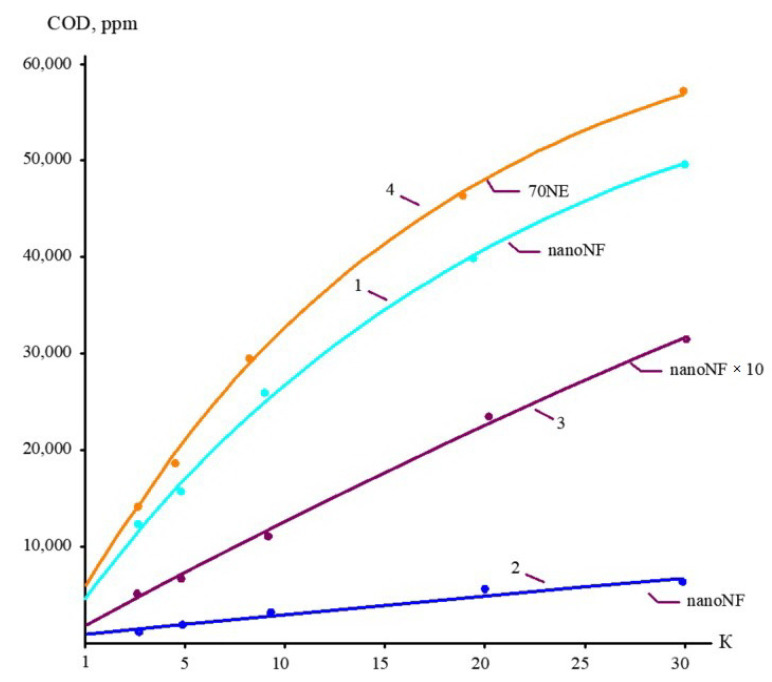
The dependencies of COD in permeates and concentrates on K: 1—first stage of leachate concentration using “nanoNF” membranes; 2—first stage of permeate treatment using “nanoNF” membranes; 3—first stage of leachate concentrate after dilution using “nanoNF” membranes; 4—first stage of leachate treatment using “70NE” membranes.

**Figure 8:**
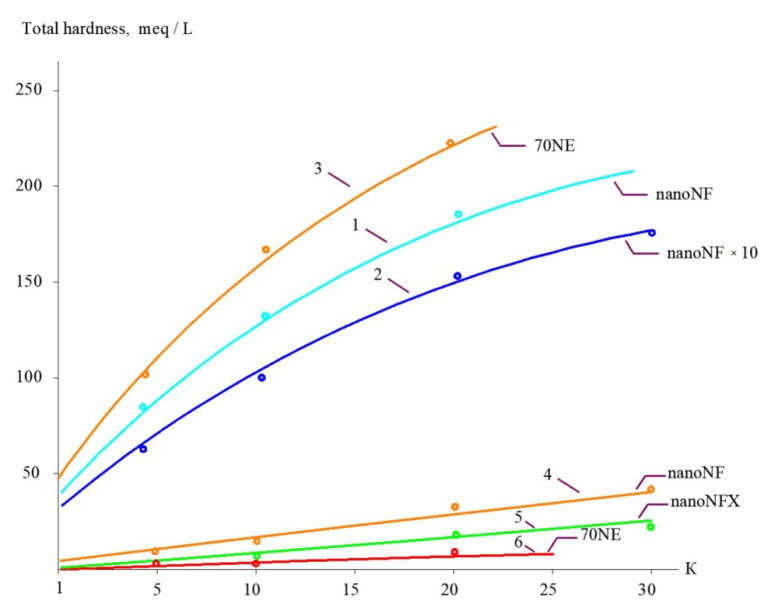
The dependencies of calcium concentration in permeates and concentrates on K: 1—landfill leachate treatment in the first stage using “nanoNF” membranes; 2—second stage of concentration using “nanoNF” membranes after dilution; 3—landfill leachate treatment in the first stage using “70NE” membranes; 4—first-stage permeate using “nanoNF” membranes; 5—second-stage permeate using “nanoNF” membranes after dilution; 6—first-stage permeate using “70 NE” membranes.

**Figure 9:**
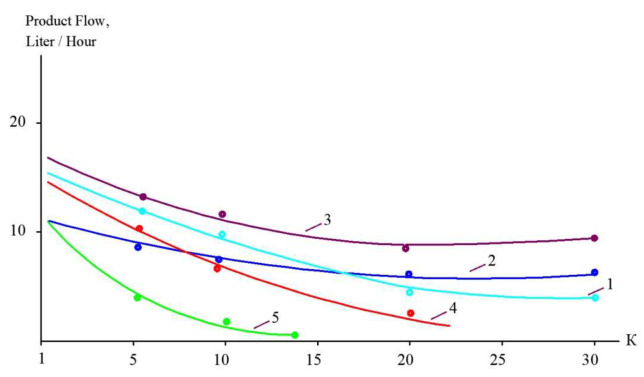
Reduction in membrane-specific product flow as a function of K for treatment of different landfill leachates with “70 NE” membranes (1, 3); “nanoNF” membranes (2, 4); SW membranes (5).

The concentrations of all species contained in leachate (total salt content; COD; alkalinity; and concentrations of calcium ions, ammonia, and other contaminants) of the landfill “Voskresensk” as a function of the initial volume reduction coefficient K (defined as a ratio of leachate flow rate to concentrate flow rate) are presented in Figure 6, Figure 7, Figure 8 and Figure 9.

During the operation of the test unit (Figure 5) in circulation mode, an increase in the TDS and COD was observed (Figure 5 and Figure 6). The dissolved organics accumulated in the concentrate significantly increased the osmotic pressure and reduced the product flow (Figure 6). A low salt rejection of nanofiltration “nanoNF” membranes allows the penetration of salts in the permeate, which results in a small difference in TDS in the concentrate and permeate. This allows us to achieve high TDS values in the concentrate (up to 120–160 g per liter) at relatively low values of working pressures (no higher than 1.6–1.8 MPa). Nanofiltration membranes in the first stage provide a relatively high rejection of organics (90%), which results in the growth of COD values in the first-stage concentrate [22,23]. Sodium and ammonium penetrate through the low-rejection membrane into the first-stage permeate stream. The first-stage permeate is treated in the second membrane stage using “nanoNF” membranes that provide the complete removal of organics. The second-stage permeate is also used to dilute the first-stage concentrate for further treatment in the third stage to separate organics and salts. The COD value of the third-stage concentrate can reach 120–160 g/L [18,32,33].

### 3.2. Separation Results

**Figure 10:**
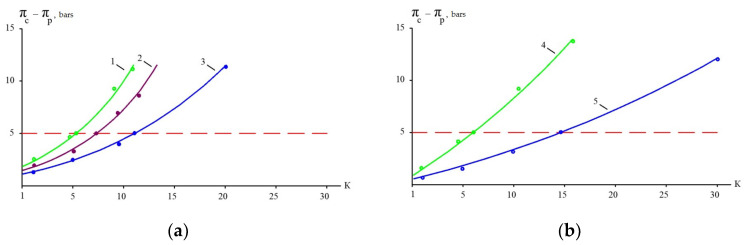
The dependencies of the working osmotic pressure on K: (**a**) experiments with “70NE” membranes; (**b**) experiments with “nanoNF” membranes. 1, 4—the first cycle of landfill leachate concentration; 2, 5—the second cycle of concentration after dilution; 3—the third cycle of concentration after the second dilution.

**Figure 11:**
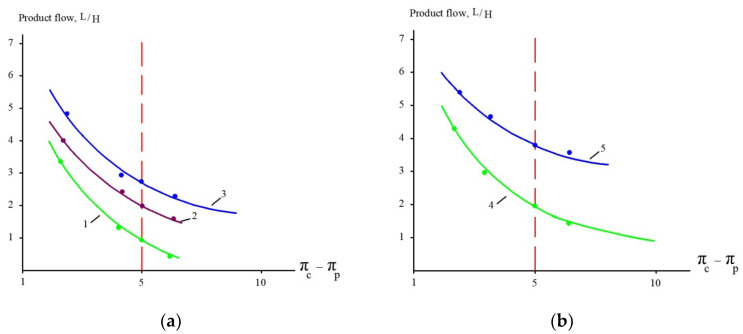
Product flow as a function of osmotic driving force: experiments with (**a**) “70NE” and (**b**) “nanoNF” membranes. 1, 4—the first cycle of landfill leachate concentration; 2, 5—the second cycle of concentration after dilution; 3—the third cycle of concentration after the second dilution.

The results of experiments conducted to separate organics and salts from the concentrate and to reduce the concentrate flow during landfill leachate treatment are presented in Figure 10 and Figure 11. “NanoNF” membranes allow the efficient separation of organics and salts to achieve a high ratio of COD to TDS in two dilution and concentration cycles. As can be seen in Figure 10 and Figure 11, “70NE” membranes demonstrated a lower ability to increase the ratio. This is attributed to a higher rejection of salts by “70NE” membranes, and it allowed us to develop a new technology to reduce both concentrates due to the separation of organics and salts.

### 3.3. Calcium Carbonate Scaling and Supersaturation

**Figure 12:**
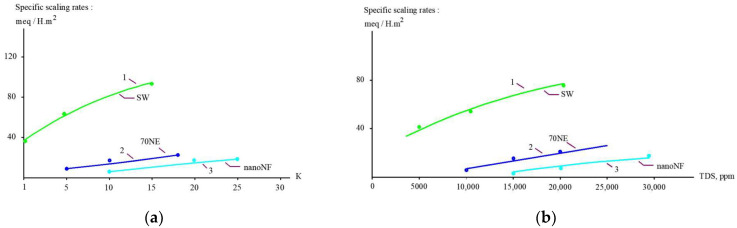
Comparison of scaling rates versus (**a**) K and (**b**) TDS. 1—“SW” membranes”; 2—“70 NE” membranes; 3—“nanoNF” membranes.

**Figure 13:**
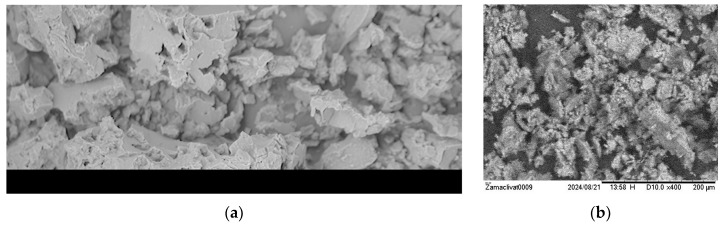
Foulant removed from the membrane surface after autopsies of SW (“seawater”) high-pressure membrane modules operated at the “Voskresensk” landfill: (**a**) flakes of sediment covered by an organic layer under a microscope; (**b**) a SEM photo of the sediment.

**Figure 14:**
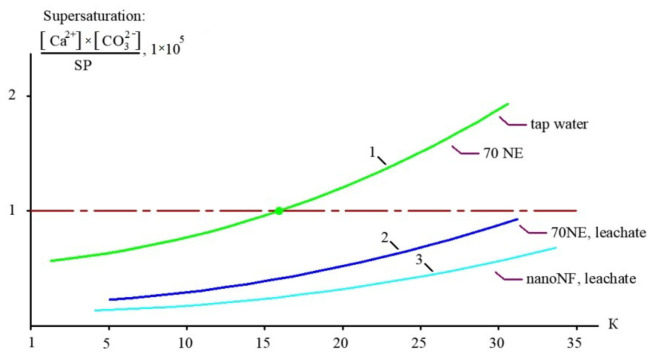
The dependencies of calcium carbonate supersaturation on K under different conditions: 1—treatment of Moscow tap water using “70 NE” membrane; 2—treatment of the “Voskresensk” landfill leachate using “70 NE” membrane; 3—treatment of the “Voskresensk” landfill leachate with “nanoNF” membrane.

**Figure 15:**
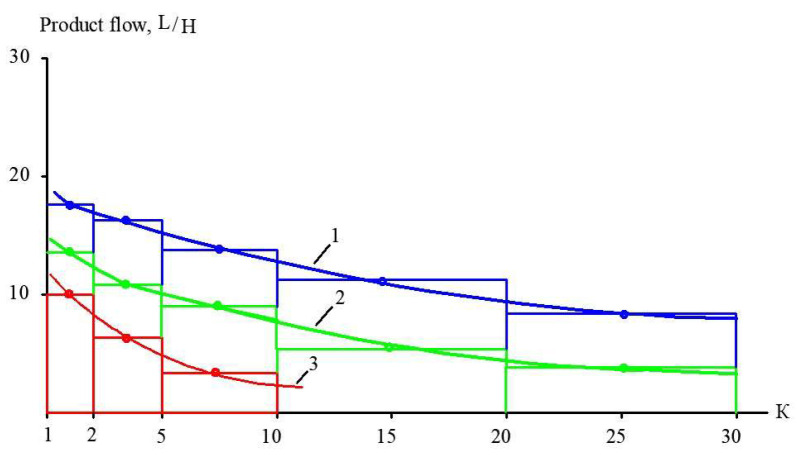
Techniques of membrane surface evaluation required to achieve the desired coefficient K during landfill leachate treatment in the first stage (Figure 9): 1—“nanoNF” membranes; 2—“70 NE” membranes; 3—“SW” high-pressure RO membranes.

Figure 12, Figure 13 and Figure 14 present the results of the evaluation of both the calcium carbonate scaling rates in membrane modules tailored with nanofiltration membranes and the nucleation rates in concentrate flow. The main steps to evaluate scaling rates are described in [20,25]. Scaling rates were determined throughout the test runs that were performed using the membrane test unit shown in Figure 5. As it was claimed in our earlier research [25], nucleation occurs in the “dead areas” on the membrane surface where supersaturation is maximized. This maximum was defined as 1 × 10^5^ [25] and was evaluated based on the results of SEM investigations [20,24,25]. As can be seen in Figure 12 and Figure 14, the use of nanofiltration membranes for leachate treatment does not provide favorable conditions for calcium carbonate scaling and demonstrates very low scaling rates. On the contrary, high working pressures and high-rejection membranes create favorable conditions for calcium carbonate nucleation and crystallization [20,24,25]. Curve 3 in Figure 12 is built based on the determination of scaling rates in membrane modules tailored with high-pressure membranes [20,25]. Figure 12 and Figure 14 show graphs of the scaling rate versus K and TDS when “Voskresensk” landfill leachate is treated with seawater membranes under high pressure (Figure 13 and Figure 15), which was taken from our previous research conducted in 1990 and devoted to calcium sulfate and calcium carbonate formation in RO modules with high rejection at high pressures [20]. The conventional use of seawater high-pressure RO membranes for leachate treatment is clearly the main reason for rapid membrane fouling, the rapid increase in flow resistance, and the reduction in product flow. This reduction in membrane permeability is usually mistakenly attributed to the high COD of the leachate. Autopsies of membrane elements used for “Voskresensk” leachate treatment demonstrated organic deposits on the membrane surface. Contrary to the concerns of the experts, the main obstacle in safe membrane operation during leachate treatment is attributed not to organic fouling and not to high COD values of feed water, but to calcium carbonate formation “hidden” in the organic fouling layer and formed due to supersaturation conditions provided by high pressures and high-rejection membranes in membrane channels of “seawater” membrane modules. However, the SEM element analysis that was undertaken revealed large amounts of calcium present in deposits (Figure 13). This confirms that calcium carbonate is the main foulant to deal with, and the use of nanofiltration membranes can change the reliability of membrane facility performance and reduce operational costs. These results enable us to determine the maximum permitted recovery at which homogeneous nucleation begins in the concentrate flow.

Figure 14 shows the dependencies of supersaturation values in concentrates (the ratio of the product of calcium and carbonate ion concentrations to the Solubility Product) on the initial flow reduction coefficient K (which represents the ratio of the feed flow to concentrate flow). Table 4 presents the results of calculations of the supersaturation ratios achieved in the concentrate flow at high recoveries for leachate, and those of tap water treated with “70 NE” membranes are shown for comparison. This function is crucial for knowing when the recovery value is selected. The beginning of scale deposition at high K values is more likely attributed to the achievement of supersaturation in the flow. Table 4 demonstrates the results of calculating supersaturation values achieved in the concentrate flow during the experiment. The beginning of homogeneous nucleation in the flow corresponds to a supersaturation value of 1.1 × 10^5^ [25].

### 3.4. Organic Fouling

Organic fouling of membranes was extensively investigated and the adhesive abilities of membranes were evaluated [29,30,31]. Harvey Winters [31] concluded that NOM caused membrane fouling due to the use of flocculants, which caused flocculation and formed particles that caused fouling and reduced membrane product flow. Humic acid molecules [31] are polyanions and form hydrogen bonds with water molecules. Thus, their solutions are stable even at high concentrations. They form a thin layer that does not significantly influence membrane performance. The main problem of organic fouling is the use of cationic polymers to start coagulation.

After the surface is covered with an adsorption layer of organics (adsorption film), the adhesion rate of organics decreases as the number of organic molecules layers increases. As it was determined throughout experiments aiming to accumulate organics, the amount of organics did not exceed 1000–1100 mg per square meter of membrane. After accumulating this amount, the adhesion rate decreased [30,31]. Thus, we control organic fouling by cleaning the membrane using caustic solution after 1500–1600 mg is collected on one square meter [31].

The fouling of organics that is represented by high COD values was investigated in a number of previously published articles [25,29,30,31]. The leachate organics are destruction products that are poorly adsorbed on the membrane surface compared with NOM (natural organic material) that forms the color of natural water [25,30]. Rates of organic fouling during treatment of landfill leachate were determined based on the mass balance of organics (COD values) [19,20,24].

### 3.5. Processing of Results

**Figure 16:**
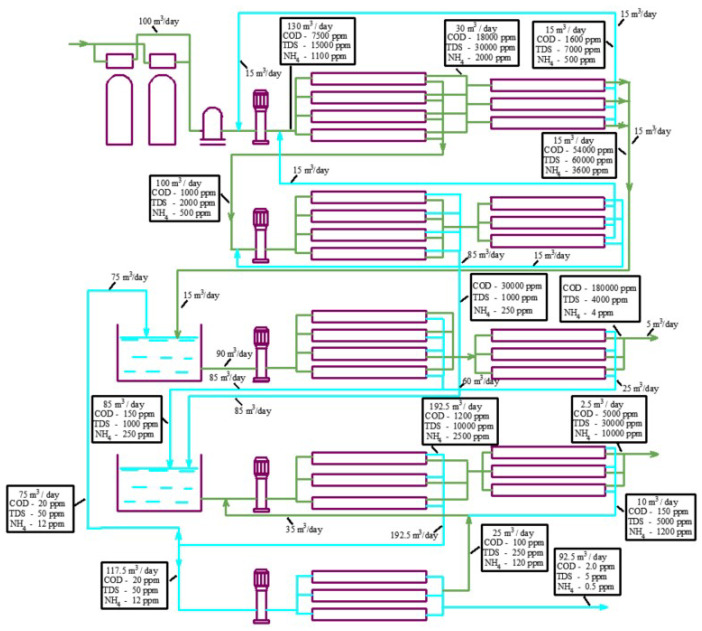
A flow balance diagram to separate the concentrate into organic and salt solutions using the developed technology: 1—pretreatment filters; 2—cartridge filter; 3—working pumps for the first, second, third, fourth, and fifth stages; 4—first stage of nanofiltration membranes “nanoNF”; 5—first stage of concentrate reduction using membrane; 6—second stage of nanofiltration using “nanoNF membranes”; 7—second stage of concentrate reduction using membrane; 8—concentrate dilution tank; 9—third stage of nanofiltration using “nanoNF” membranes; 10—third stage of concentrate reduction using membrane to produce concentrated organic solution; 11—permeate collection tank as feed water for the fourth membrane stage; 12—fourth stage using low-pressure RO membranes; 13—fourth stage of concentrate reduction using membrane; 14—fifth stage using low-pressure RO membranes.

Figure 15 shows the main techniques to evaluate the membrane surface and the number of membrane spiral wound elements required to achieve the designed value of coefficient K in each membrane stage of the technological scheme. Using the two ranges of K (from 1 to 5 and from 5 to 10), we select an average product flow within each range. For the flow diagrams shown in Figure 1 and Figure 16, we carry out calculations for each membrane stage based on the experimental results shown in Figure 9, Figure 10 and Figure 11. The evaluated number of membrane elements needed in each stage allows us to calculate the annual amount of cleaning chemicals to remove organic and inorganic foulants. In addition, the number of membrane spiral wound elements provides us with the annual costs of membrane replacement [29,30]. Electric power consumption is evaluated assuming a working pressure of 16 bars in each stage and flow rates of the working pumps. The comparison of the main characteristics of technological schemes presented in Figure 1 and Figure 16 (annual power consumption, annual chemical consumption, annual membrane replacement costs, and annual concentrate amount) is presented in Table 5. Calculations are made for the facility with 100 cubic meters of landfill leachate capacity. The leachate composition corresponds to the “Voskresensk” landfill (Table 1).

### 3.6. Industrial Application of the Results

To reach high recoveries and to separate organics and salts, a new flow diagram was developed by the authors (Figure 15). Initial landfill leachate (flow rate of 100 m^3^/day; COD of 7500 mg/L; TDS concentration, expressed as NaCl, of 15,000 mg/L) enters the membrane unit and passes through first-stage modules 4 and 5 furnished with nanofiltration low-rejection membranes (average salt rejection varies from 50 to 70%). After the first stage, we obtain a permeate solution with a COD value of 500 mg/L and a NaCl content of 1500 mg/L. The concentrate flow rate is reduced to 3 m^3^/day and the permeate flow rate is 97 m^3^/day. The permeates obtained in the first and third stages that contain organics are treated in the second stage using low-rejection nanofiltration membranes 6 and 7. The second- and first-stage concentrates are forwarded to mixing tank 8. The second-stage permeate is also forwarded to mixing tank 5 to dilute the concentrate and separate the organics.

The concentrate is diluted to a ratio of 1 to 5 in mixing tank 8. Since the membrane rejects organics better than monovalent ions, the ratio of organic to salt concentrations in the mixture is higher than in the feed water (leachate). After dilution in tank 8, the mixture concentrates in modules 9 and 10 to reach a maximum COD value. The third-stage permeate is forwarded to tank 11 and further directed using pump 3 to fourth-stage modules 12 and 13, tailored by nanofiltration membranes. The concentrate of the fourth-stage modules that contains the organic residues is returned to tank 8, and the permeate of the fourth stage is directed to the first-stage reverse osmosis modules 12 to produce deionized water. The concentrate after the first-stage reverse osmosis modules 12 is further concentrated by a “cascade” of nanofiltration modules 13 to achieve the TDS value of 140 g/L.

After the first reverse osmosis desalination stage (module 9), the purified water passes through one more stage of treatment to reduce the ammonium concentration. Concentrates from each stage are returned to the inlet of the previous stage. The final product water has a TDS value of 5–10 ppm and an ammonium concentration of about 0.2 ppm. Thus, the landfill leachate is separated into the final water product and two concentrates: saline concentrate with a salt content of 100–150 g/L (a mixture of sodium salts and ammonium) and organic concentrate (a mixture of organics (with a COD concentration of 100 g/L) and calcium, magnesium, sodium, and ammonium salts with a concentration of 30–40 g/L). The saline concentrate flow rate is equal to 2% of the initial leachate flow rate, and the organic solution concentrate is 1% of the initial leachate flow rate. The organic concentrate can be mixed with soil or with dewatered sludge from natural and wastewater treatment plants and used for landfill reclamation [18,19]. The salt concentrate can also be evaporated to dry salts or used as a raw material in fertilizer production [19].

For the leachate solution with a COD value of 7500 mg/L and a TDS value of 15,000 ppm, technical and economic calculations were carried out to compare the operating costs of traditional high-pressure membranes to the new technology [19,33]. Calculations of the annual operating costs for landfill leachate treatment and concentrate utilization using different technological schemes are given in [34,35]. The developed technology provides lower concentrate amounts and substantial savings spent on concentrate disposal and utilization [36].

## 4. Conclusions

The experimental results showed that the “nanoNF” membranes with low rejection had the best effect on leachate separation. Due to the difference in rejection for salts and organic matter, we were able to achieve a 10-fold increase in the COD/TDS ratio in two concentrating and dilution cycles. In addition, the use of low-pressure membranes showed a reduction in calcium carbonate scaling. A new modification of the spiral wound membrane module with “open channels” was proposed for landfill leachate treatment, which uses “nanoNF” low-rejection membranes. This makes it easier to simplify pretreatment and reduce operational costs. The potential to reduce the concentrate flow by 30 times allowed us to avoid evaporation. The RO concentrate flow reduction technology requires a pressure of no more than 18 bars, which saves operational costs compared to the use of evaporation.

The main results obtained are as follows:

- The determined scaling and organic fouling rates are presented and illustrated by SEM photos and spectral analysis performed after membrane autopsies;
- The investigation demonstrated that the formation of calcium carbonate is excluded when using nanofiltration membranes;
- The economical calculations demonstrated the advantages of the new technology based on the use of low-rejection membranes to reduce concentrate flow as compared to conventional high-pressure membrane applications.

## Figures and Tables

**Table 1:** Chemical compositions of leachates delivered from different landfills in Moscow region, used in experiments.

Parameters	Landfill 1 (“Timokhovo”)	Landfill 2 (“Aleksandrov”)	Landfill 3 (“Voskresensk”)
Suspended solids, ppm	220	329	—
COD, ppm	20,000	4387	7500
BOD, ppm	915	2690	—
Ammonium (NH_4_^+^), ppm	2400	380	1100
Nitrates (NO_3_^−^), ppm	59	—	—
Alkalinity, ppm	13,546	760	760
Chlorides (Cl^−^), ppm	2700	980	—
Sulfate (SO_4_^2−^), ppm	1200	86	860
Total hardness, milliequivalents/liter	20	5.5	27
pH	8.9	7.7	—
Na^+^+K^+^, ppm	1000	—	—
Phenols, ppm	0.2	—	—
TDS, ppm	9000	3580	15,000

**Table 2:** Results of calculating the minimum working pressure required to purify leachates and achieve the desired recovery value.

Investigated Solution Parameters	Parameters	Feed Water ppm/π, bars	Permeate, ppm/bars		Concentrate, ppm/bars		Permeate, ppm/bars		Concentrate, ppm/bars	
			K = 3	K = 9	K = 3	K = 9	K = 20	K = 30	K = 20	K = 30
Landfill N 1 “Timokhovo” Solution 1	NaCl	15,000/10	5000/3.5	11,000/8	25,000/18	35,000/25	—	—	—	—
	COD	20,000/3.0	2000/0.3	6000/0.9	56,000/9	180,000/25	—	—	—	—
	π	13.0	3.83	8.9	27	50	—	—	—	—
Landfill N 1 “Timokhovo” Solution 2 (after dilution 1:3)	NaCl	8000/5.5	3000/2.0	5000/3.3	15,000/10	25,000/18	—	—	—	—
	COD	20,000/3.0	2000/0.3	5000/0.8	55,000/9	150,000/22	—	—	—	—
	π	8.5	2.3	4.1	19	30	—	—	—	—
Landfill N 1 “Timokhovo” Solution 3 (after dilution 1:10)	NaCl	2500/1.8	8000/0.6	15,000/1.1	4500/3	8000/5.1	—	—	—	—
	COD	15,000/2.2	1500/0.22	4500/0.66	45,000/6.7	120,000/18	—	—	—	—
	π	4.0	0.82	1.76	9.7	23.1	—	—	—	—
Landfill N 2 “Aleksandrov” Solution 1	NaCl	4000/2.6	1300/500	—	7000/4.5	11,000/6.8	—	—	17,000/11	—
	COD	5000/0.8	—	—	14,000/2.1	27,000/4	—	—	59,000/9.5	—
	π	3.4	—	—	6.6	10.8	—	—	20.5	—
Landfill N 2 “Aleksandrov” Solution 2 (after dilution 1:20)	NaCl	850/0.5	300/0.2	700/0.5	1600/1.1	2500/1.8	1000/0.7	1300/0.9	3800/2.6	4600/3.1
	COD	2600/0.4	260/0.04	750/0.11	6500/1.1	19,000/2.6	1900/0.3	2800/0.45	38,000/5.2	54,000/9.0
	π	0.9	0.24	0.65	2.2	4.4	1.0	1.35	7.8	12.1

**Table 3:** Calculation results of osmotic pressure during the concentration of leachate taken from the “Voskresensk” landfill.

Ratio: Concentration, Gram/Liter; Osmotic Pressure, Bars														
Investigated Solution Parameters	Parameters	Feed Water	Permeate		Concentrate		Permeate		Concentrate		Osmotic Pressure Difference, $\pi_{c}$ − π_p_			
			K = 3	K = 9	K = 3	K = 9	K = 20	K = 30	K = 20	K = 30	K = 3	K = 9	K = 20	K = 30
Solution 1	NaCL, g/L	15/10	5.0/5	7/7.0	30/20	60/40	—	—	—	—	15	33	—	—
	COD, ppm	7500/1.0	1000/0.12	1600/0.2	18,000/2.5	69,000/9.0	—	—	—	—	2.38	8.8	—	—
	π total, bars	9.0	6.12	7.2	22.5	49,0					17.38	41.8		
Solution 2 (after dilution 1:10), K = 3	NaCL, g/L	3.0/2.0	1.0/0.7	1.5/1.0	6.0/4.0	12/8.0					3.3	7.0		
	COD, g/L	1800/0.25	180/0.02	400/0.05	5000/0.75	14,000/2.0					0.73	1.95		
	π total, bars	2.25	0.72	1.05	4.75	10.0					4.03	8.95		
Solution 3 (after next dilution 1:10), K = 9	NaCL, g/L	1.2/0.8	0.5/0.35	0.8/0.5	2.4/1.6	4.8/3.2	1.2/0.8	1.8/1.2	10/7	12/9	1.25	2.7	6.2	5.8
	COD, g/L	1400/0.2	140/0.016	300/0.03	4000/0.6	11,000/1.7	400/0.6	800/0.12	21,000/3.1	30,000/5	0.584	1.67	2.5	4.86
	π total, bars	1.0	0.36	0.53	2.2	4.9	0.86	1.32	10.1	14	1.84	4.37	9.24	12.68
Solution after separation	NaCL, g/L: 75 L	20/1.3	3.1/0.2	15/0.12	40/2.6	70/4.8	—	—	—	—	1.5	4.68		
	COD, g/L: 25 L	30,000/4.0	3000/0.4	6000/0.8	85,000/11	240,000/30	—	—	—	—	10.6	29.2		

**Table 4:** Results of supersaturation calculations.

Investigated Membrane Models	K	Ca meq/L	HCO_3_ meq/L	pH	TDS ppm	$\frac{\left[{\mathrm{CO}}_{3}^{2-}\right]}{[\mathrm{HC}O_{3}^{-}]}$	[CO_3_^2−^] ppm	$\frac{\left[{\mathrm{Ca}}^{2+}]\times [{\mathrm{CO}}_{3}^{2-}\right]}{\mathrm{SP}}$
First stage of leachate treatment using “nanoNF” membranes	5	81	—	6.8	21,000	—	—	0.1
	10	116	920	7.0	30,000	0.011	0.04	0.11
	20	160	1200	7.2	36,000	0.018	0.1	0.17
	30	190	1400	7.4	42,000	0.02	0.14	0.31
	35	196	1550	7.5	47,000	0.03	0.15	0.36
First stage of leachate treatment using “70NE” membranes	5	100	—	—	—	—	—	0.24
	10	160	—	—	—	—	—	0.3·10^5^
	20	220	—	—	—	—	—	0.6·10^5^
	30	240	—	—	—	—	—	0.8·10^5^
	35	250	—	—	—	—	—	1·10^5^
Treatment of Moscow tap water using “70NE” membranes	5	8	—	7.5	—	—	—	0.72
	10	15.5	—	8.4	—	—	—	0.8
	20	26	—	9.5	—	—	—	1.3
	30	40	—	9.6	—	—	—	2.0
	35	48	—	9.7	—	—	—	—

**Table 5:** Comparison of different membrane behaviors and operational costs.

Operational Characteristics	Seawater RO Membranes Followed by Ultrahigh-Pressure RO (Figure 1a)	Nanofiltration Membranes in the First and Second Stages Followed by Low-Pressure RO Stage (Figure 1b)	Nanofiltration Membranes in the First and Second Stages Followed by Organics–Ion Separation Stages (Figure 16)
Specific power consumption: kWh m^−3^	19	3.6	4.8
Total number of 8040 spiral wound membrane elements PCS	61 49—SWRO; 12—Ultrahigh-pressure RO	42 (NF membranes and RO membranes)	56
Annual number of cleanings	8	8	8
Annual consumption of cleaning chemicals, kg	192	174	192
Antiscalant dose, ppm	10	—	—
Total annual antiscalant consumption, kg	500	—	—
Concentrate flow, m^3^/hour	15.0	5.0	2.5

## Data Availability

The data are available in publications.
